# Managing the risk of circulating vaccine-derived poliovirus during the endgame: oral poliovirus vaccine needs

**DOI:** 10.1186/s12879-015-1114-6

**Published:** 2015-09-24

**Authors:** Radboud J. Duintjer Tebbens, Kimberly M. Thompson

**Affiliations:** Kid Risk, Inc., 10524 Moss Park Rd., Ste. 204-364, Orlando, FL 32832 USA

## Abstract

**Background:**

The Global Polio Eradication Initiative plans for coordinated cessation of oral poliovirus vaccine (OPV) use, beginning with serotype 2-containing OPV (i.e., OPV2 cessation) followed by the remaining two OPV serotypes (i.e., OPV13 cessation). The risk of circulating vaccine-derived poliovirus (cVDPV) outbreaks after OPV cessation of any serotype depends on the serotype-specific population immunity to transmission prior to its cessation.

**Methods:**

Based on an existing integrated global model of poliovirus risk management policies, we estimate the serotype-specific OPV doses required to manage population immunity for a strategy of intensive supplemental immunization activities (SIAs) shortly before OPV cessation of each serotype. The strategy seeks to prevent any cVDPV outbreaks after OPV cessation, although actual events remain stochastic.

**Results:**

Managing the risks of OPV cessation of any serotype depends on achieving sufficient population immunity to transmission to transmission at OPV cessation. This will require that countries with sub-optimal routine immunization coverage and/or conditions that favor poliovirus transmission conduct SIAs with homotypic OPV shortly before its planned coordinated cessation. The model suggests the need to increase trivalent OPV use in SIAs by approximately 40 % or more during the year before OPV2 cessation and to continue bOPV SIAs between the time of OPV2 cessation and OPV13 cessation.

**Conclusions:**

Managing the risks of cVDPVs in the polio endgame will require serotype-specific OPV SIAs in some areas prior to OPV cessation and lead to demands for additional doses of the vaccine in the short term that will affect managers and manufacturers.

## Background

The Global Polio Eradication Initiative (GPEI) primarily relied on oral poliovirus vaccine (OPV) to make great progress toward interrupting all wild poliovirus (WPV) transmission, including apparent interruption of indigenous serotype 2 WPV (WPV2) by the year 2000 and serotype 3 WPV (WPV3) by the year 2012 [1–3]. OPV contains attenuated live poliovirus that can infect both recipients and contacts, mimicking infection with WPV to provide good intestinal immunity with a very low risk of vaccine-associated paralytic polio [4, 5]. However, in places with very low population immunity to transmission, OPV can evolve to acquire WPV-like properties and cause outbreaks of circulating vaccine-derived poliovirus (cVDPV) [6–8]. The risks associated with OPV motivate plans for globally-coordinated cessation of all serotype 2 - containing OPV (i.e., OPV2 cessation) in 2016 and the remaining two OPV serotypes after 2018 (i.e., OPV13 cessation) [2]. Dynamic poliovirus transmission and OPV evolution models based on the current state of the evidence [7, 9–12] strongly suggest the need to achieve high homotypic population immunity to transmission at the time of OPV cessation of any serotype to prevent OPV-related viruses from evolving into cVDPVs shortly after OPV cessation [13].

Clinical trials suggest that the inactivated poliovirus vaccine (IPV) provides excellent humoral immunity to protect from paralytic poliomyelitis disease and also effectively boosts intestinal immunity in individuals with prior immunity induced by a live poliovirus infection (i.e., WPV, OPV, OPV-related, or VDPV) [10, 14–16]. However, immunity induced by IPV-alone does not protect as well as OPV from asymptomatic participation in fecal-oral poliovirus transmission and does not provide any secondary immunity to contacts [10, 17, 18]. In populations with conditions conducive to fecal-oral poliovirus transmission, IPV use thus provides little reduction in potential participation in poliovirus transmission among previously unvaccinated individuals that contribute most to transmission [19–21]. Given that cVDPVs remain most likely to emerge in places with low routine immunization (RI) coverage and intense fecal-oral transmission, models that factor in the higher immunogenicity and intestinal boost provided by IPV [12] suggest that IPV use does not appear to substantially reduce the risk or consequences of cVDPV emergences from parent OPV strains or already circulating partially- or fully-reverted OPV-related viruses following OPV cessation [21–23]. Consequently, maximizing population immunity just prior to OPV cessation of any given serotype(s) requires high homotypic OPV use up until OPV cessation [13, 21].

While RI with OPV continues to rely on trivalent OPV (tOPV), which contains all 3 serotypes and currently represents the only licensed OPV vaccine containing serotype 2, the GPEI focus on eradicating the remaining two WPV serotypes led it to shift many supplemental immunization activities (SIAs) since 2005 from tOPV to monovalent OPV serotype 1 (mOPV1), monovalent OPV serotype 3 (mOPV3), and bivalent OPV (bOPV, serotypes 1 and 3) [24]. However, to manage the risks of serotype 2 cVDPVs (cVDPV2s) after OPV2 cessation (i.e., prevent their creation), the GPEI needs to ensure sufficient use of tOPV prior to OPV2 cessation. After OPV2 cessation, the vaccination strategy should maintain high vaccination intensity, but with bOPV instead of tOPV, until OPV13 cessation. Given the lead times associated with vaccine orders, timely planning of OPV needs prior to OPV cessation represents a key part of risk management. We seek to characterize the expected needs for the different OPV formulations leading up to OPV cessation using an integrated global model of long-term poliovirus risk management policies for a strategy that supports an expectation of no cVDPVs following OPV cessation [25]. We focus on characterizing expected vaccine needs for the current global plans and timelines for OPV cessation for the GPEI Strategic Plan 2013-2018 [2]. We separately consider the implications of tOPV vs. bOPV choices for meeting WPV eradication goals and managing cVDPVs [26].

## Methods

We used an existing integrated global model of long term poliovirus risk management policies (i.e., the global model) [25] that relies on a differential equation - based dynamic poliovirus transmission and OPV evolution model [9, 12] to simulate poliovirus spread and immunity within populations and cVDPV emergence in the event of insufficient population immunity to transmission. Specifically, given that susceptible individuals remain infectious with a poliovirus for approximately 30 days [12] and that the model assumes that any OPV-related virus introduced prior to OPV cessation can continue to transmit as long as its prevalence remains above a certain threshold, it takes some time before OPV-related viruses die-out after OPV cessation. If during that time population immunity to transmission drops to low enough levels, then the OPV-related viruses will continue to transmit and ultimately result in a cVDPV outbreak [13]. The model simulates OPV cessation as planned by the GPEI [2] with OPV2 cessation on April 1, 2016 as currently targeted, [27] followed by OPV13 cessation on April 1, 2019, which remains within the current window for this event [2]. The global model does not characterize actual individual countries, which vary widely in size and immunization and exposure histories, but instead uses generalized approximations of national-level demographic [28] and vaccine coverage data [29] for 710 subpopulations of roughly equal size averaging approximately 10 million people (as of 2013), grouped into 71 blocks of 10 subpopulations that mix preferentially with each other. We include 4 blocks with conditions like the last WPV-endemic areas in the world, including the presence of an under-vaccinated subpopulation with very low immunization rates in each [9, 19, 30] We stratify the 71 blocks by World Bank income level (i.e., low, lower middle, upper middle, and high) [31] and polio vaccine use (i.e., OPV-only, sequential IPV/OPV use, IPV-only) [32] according to the distribution of the population as of 2013 [28].

To approximate levels of population immunity to transmission at the beginning of the analytical time horizon in 2013 with a reasonable simulation run time, the global model specifies an accelerated and generalized run-up of 43 years using a simplified history of polio vaccination in each subpopulation [25]. The true vaccination history in different countries remains highly complex and difficult to reconstruct, particularly as it relates to SIAs [33]. Current SIA schedules for polio depend on many factors, including the epidemiological situation, financial constraints, shifting priorities, and national preferences. They remain highly irregular and sometimes involve different activities on a small scale, in some cases implying heterogeneity at a finer level than the 10 million people we use to characterize subpopulations in the model. The global model abstracts from this complexity by specifying SIA schedules for each subpopulation that depend on the assumed coverage with 3 or more non-birth RI doses (POL3), basic reproduction number (R_0_), and epidemiologic state (i.e., before eradication of all WPVs, after eradication of all WPVs, or during an ongoing outbreak of cVDPV or imported WPV). Although the global model does not use actual historic and planned immunization activities, from 2010 forward (i.e., the time of bOPV adoption for some SIAs), the global model aims to closely represent the true global intensity of vaccination used by countries and the GPEI.

The global model assumes sufficient frequency of tOPV SIAs to prevent cVDPV2 emergence after OPV2 cessation. This involves tOPV intensification relative to the prior frequency of tOPV SIAs starting on January 1, 2015 until OPV2 cessation on April 1, 2016. However, in some settings the need to intensify tOPV use prior to OPV2 cessation remains a topic of discussion. For this analysis, we consider scenarios without such tOPV intensification to show the potential impact on cVDPV2 risks and resulting serotype 2 monovalent OPV (mOPV2) needs for outbreak response. Given our focus on characterizing the difference in cVDPV2 risks with or without tOPV intensification in the short-term, we consider a short time horizon and ignore all long-terms risks of poliovirus reintroductions (i.e., immunodeficiency-associated vaccine-derived poliovirus introductions and other releases that stochastically occur over the long-term considered in detail in the global model [25]).

Table 1 shows the assumed preventive SIA schedules for subpopulations in blocks that previously interrupted indigenous WPV transmission. While Table 1 does not include the schedule for the small number of blocks that did not yet interrupt indigenous WPV transmission or blocks experiencing cVDPV or WPV importation outbreaks [25], it captures the SIA assumptions for the majority of blocks. We compared our estimates of required OPV doses associated with intensification efforts to the GPEI SIA plans as of February 1, 2015. As shown in Table 1, we determine the annual number of SIAs based on modeled RI coverage and R_0_ (i.e., we differentiate between settings with very high R_0_ > 10 and lower R_0_). During tOPV intensification, we use tOPV for all SIAs in subpopulations with 1 annual SIA, for the first 2 annual SIAs for subpopulations with at least 3 annual SIAs, and for at least the first 3 annual SIAs for subpopulations with 5 or more annual SIAs. We allow the model to maximize population immunity at the time of OPV2 cessation by optimally using tOPV in the run-up to OPV2 cessation. Thus, the model uses tOPV in all SIAs conducted in 2016 that occur before the OPV2 cessation date of April 1, 2016. In addition to tOPV intensification before OPV2 cessation and consistent with the current strategy, [2] the model further assumes that all countries that used OPV-only as of 2013 incorporate IPV into their RI schedules starting January 1, 2015, either as a co-administered dose with the 3^rd^ non-birth RI OPV dose (in low- and lower middle-income blocks) or as the first two doses in a sequential IPV/OPV schedule (in upper middle-income blocks).

**Table 1:** Assumptions for planned, preventive SIAs (pSIAs) through OPV2 cessation in OPV-using blocks that interrupted indigenous wild poliovirus transmission (adapted from Thompson and Duintjer Tebbens (2015) [26])

Time period	RI coverage (*POL3*)	SIA schedule showing: vaccine (day(s) of year)
Before tOPV intensification on January 1, 2015	0.05 or 0.1	tOPV (0, 40); bOPV (80, 140, 240, 300)
	0.3	tOPV (0, 40); bOPV (80, 140, 240)
	0.6 (R_0_ ≤ 10)	tOPV (0); bOPV (60, 120)
	0.6 (R_0_ > 10)	tOPV (0, 40); bOPV (80, 140, 240)
	0.9	tOPV (0)
	0.98 (R_0_ ≤ 10)	No SIAs
	0.98 (R_0_ > 10)	tOPV (0)
During tOPV intensification (January 1, 2015 to April 1, 2016)	0.05 or 0.1	tOPV (0, 40, 80, 300); bOPV (140, 240)
	0.3	tOPV (0, 40, 80); bOPV (140, 240)
	0.6 (R_0_ ≤ 10)	tOPV (0, 60); bOPV (120)
	0.6 (R_0_ > 10)	tOPV (0, 40, 80); bOPV (140, 240)
	0.9	tOPV (0)
	0.98 (R_0_ ≤ 10)	No SIAs
	0.98 (R_0_ > 10)	tOPV (0)
After tOPV intensification (April 1, 2016 to April 1, 2017 or later)	0.05 or 0.1	bOPV (0, 40, 80, 140, 240, 300)
	0.3	bOPV (0, 40, 80, 140, 240)
	0.6 (R_0_ ≤ 10)	bOPV (0, 60, 120)
	0.6 (R_0_ > 10)	bOPV (0, 40, 80, 140, 240)
	0.9	bOPV (0)
	0.98 (R_0_ ≤ 10)	No SIAs
	0.98 (R_0_ > 10)	bOPV (0)

Table 2 shows different options of bOPV SIA frequencies we consider starting on January 1, 2017. We include scenarios that sustain the SIA frequency from before 2017 until OPV13 cessation (i.e., no reduction), reduce the annual number of SIAs by 1 (in subpopulations with POL3 = 0.98) or 2 (in subpopulations with POL3 ≤ 0.60) (i.e., medium reduction), or reduce the annual number of SIAs by up to 3 in subpopulations with 5 or more annual SIAs (i.e., large reduction). The current GPEI SIA plans [2] assume substantially reduced SIA frequency from 2017 forward, similar to the large reduction scenario.

**Table 2:** Scenarios for planned, preventive SIAs (pSIAs) with bOPV between OPV2 cessation and OPV13 cessation in OPV-using blocks

Time period	RI coverage (*POL3*)	SIA schedule showing: annual number of bOPV SIAs (day(s) of year), by SIA frequency scenario		
		No reduction	Medium reduction	Large reduction
Between OPV2 and OPV13 cessation (January 1, 2017 to April 1, 2019)	0.05 or 0.1	6 (0, 40, 80, 140, 240, 300)	4 (0, 40, 80, 240)	3 (0, 60, 120)
	0.3	5 (0, 40, 80, 140, 240)	3 (0, 60, 120)	2 (0, 60)
	0.6 (R_0_ ≤ 10)	3 (0, 60, 120)	1 (0)	1 (0)
	0.6 (R_0_ > 10)	5 (0, 40, 80, 140, 240)	3 (0, 60, 120)	1 (0)
	0.9	1 (0)	1 (0)	0
	0.98 (R_0_ ≤ 10)	0	0	0
	0.98 (R_0_ > 10)	1 (0)	0	0

To estimate vaccine needs, we compute the total number of OPV doses needed (D_OPV_) in a given year as:

$$ {\mathrm{D}}_{\mathrm{OPV}\kern0.5em =}{\mathrm{w}}_{\mathrm{ri}}\kern0.5em \times \kern0.5em {\mathrm{D}}_{\mathrm{ri}}\kern0.5em +\kern0.5em {\mathrm{w}}_{\mathrm{sia}}\kern0.5em \times \kern0.5em {\mathrm{D}}_{\mathrm{sia}} $$

where w_ri_ = effective wastage factor associated with RI

w_sia_ = effective wastage factor associated with SIAs

D_ri_ = annual tOPV doses administered in RI

D_sia_ = annual tOPV doses administered in SIAs

Although national RI schedules vary significantly, [33] the global model reflects a simplified characterization based on the most common schedules. To compute D_ri,_ we assume that all OPV-only-using blocks as of 2013 use tOPV for RI, assuming negligible impact of any very limited exceptions (e.g., Israel recently reintroduced bOPV into its RI program) [19]. We further assume that all OPV-only-using low- and lower middle-income blocks as of 2013 administer a birth dose at half of the POL3 coverage (bcov), with no birth doses administered in upper middle-income countries or areas using an IPV/OPV sequential schedule. We account for partial coverage assuming a 20 % chance that a child who did not receive at least 3 non-birth doses receives 1 non-birth dose and a 20 % chance that a child who did not receive at least 3 non-birth doses receives 2 non-birth doses (i.e., cov1 = cov2 = 0.2). Thus, for any subpopulation that uses OPV-only, the number of administered OPV doses in RI equals:

$$ {\mathrm{D}}_{\mathrm{ri}}\kern0.5em =\kern0.5em \mathrm{n}\mathrm{i}\kern0.5em \times \kern0.5em \left[\mathrm{P}\mathrm{O}\mathrm{L}3\kern0.5em \times \kern0.5em \mathrm{n}\mathrm{d}+\kern0.5em \left(1\hbox{-} \mathrm{P}\mathrm{O}\mathrm{L}3\right)\kern0.5em \times \kern0.5em \mathrm{c}\mathrm{o}\mathrm{v}1\kern0.5em \times \kern0.5em 2+\left(1\hbox{-} \mathrm{P}\mathrm{O}\mathrm{L}3\right)\kern0.5em \times \kern0.5em \mathrm{c}\mathrm{o}\mathrm{v}1+\mathrm{P}\mathrm{O}\mathrm{L}3\kern0.5em \times \kern0.5em \mathrm{bcov}\right] $$

where ni = annual number of surviving infants

POL3 = coverage with 3 or more non-birth RI doses in the subpopulation (varies by subpopulation)

nd = number of non-birth RI doses in the schedule (3 in all subpopulations)

cov1 = coverage with 1 non-birth RI dose given fewer than 3 non-birth RI doses (cov1 = 0.2 in all subpopulations)

cov2 = coverage with 2 non-birth RI doses given fewer than 3 non-birth RI doses (cov2 = 0.2 in all subpopulations)

bcov = relative coverage with birth dose compared to POL3 (bcov = 0.5 in all subpopulations with OPV-only-using low- or lower middle-income blocks and 0 elsewhere)

We assume that subpopulations using a sequential IPV/OPV RI schedule administer 2 IPV doses followed by 2 OPV doses with all partially covered children receiving only IPV, such that the computation for the number of OPV doses simplifies to:

$$ {\mathrm{D}}_{\mathrm{ri}}\kern0.5em =\kern0.5em \mathrm{n}\mathrm{i}\kern0.5em \times \kern0.5em \mathrm{P}\mathrm{O}\mathrm{L}3\kern0.5em \times \kern0.5em 2 $$

This equation applies only to those countries using an IPV/OPV schedule as of 2013 and excludes any GAVI-eligible or other countries that add IPV with the 3^rd^ OPV dose in 2015, consistent with the GPEIs current plans [2]. For the year 2016, we estimate the tOPV RI doses by prorating the total number of RI doses for the whole year assuming OPV2 cessation on April 1, 2016 and bOPV use from that point forward. Consistent with the GPEI cost calculations, [34] we compute RI vaccine demands based on the expected number of covered children. The GPEI bases its RI dose needs estimates on numbers of surviving infants similar to the UN World Population Prospects (WPP) [28] that we use in our global model [25]. The GPEI assumptions for RI include vaccine wastage factors of 1.33 for IPV, but no wastage assumption for OPV RI, because the GPEI does not budget for OPV RI [34]. Despite ambitious goals to lower wastage in RI, [35] which previously motivated us to use relatively low OPV wastage values for RI, [36] more recent estimates indicate wastage factors in low- and lower middle-income countries as high as approximately 2 (i.e., wastage rate of 50 %) [37] and this led us to assume effective wastage of w_ri, lmi_ = w_ri,low_ = 2 for low- and lower middle-income blocks. Consistent with other analyses, [25, 26] we assume a wastage factor of w_ri,high_ =1.11 (i.e., wastage rate of 10 %) for high-income blocks based on US data, [38] while we assume an intermediate wastage factor of w_ri,umi_ = 1.43 (i.e., wastage rate of 30 %) for upper middle-income blocks.

To compute D_sia_, for every SIA, we record the number of children under 5 years of age in the model at the time of the SIA, and we then sum over all SIAs that use different OPV formulations in a given year. In theory, the SIA vaccine needs depend on the actual coverage of each SIA. However, in practice the GPEI orders vaccine based on the total number of children in the target population rather than the expected number of covered children. To remain consistent with this practice, we effectively assume 100 % expected coverage for each SIA when we calculate D_sia_, although the global model uses best estimates of true coverage, which remain below 100 % for each SIA. For planned SIAs between 2012-2019, the GPEI wastage factors ranged from 1.07 (Benin, 2014) to 1.67 (Syrian Arab Republic, 2013), with the majority between 1.1 and 1.3 and an overall population-weighted average wastage factor of approximately 1.2. Our global model uses the WPP estimates of the number of children under 5 years of age [28]. However, in the context of comparisons, we observed that the GPEI assumes significantly different child population numbers in many countries when planning SIA vaccine needs. For example, in 2013 the GPEI planned national immunization days (NIDs) targeting 0-4 years olds for 45 countries. The GPEI assumed a total population of 0-4 year olds approximately 1.45 times greater than the population for the same 45 countries estimated by the WPP (i.e., 479 vs. 334 million children). Large countries that conduct many SIAs represent major contributors to this difference (i.e., India accounts for 59 million, Nigeria for 24 million, Pakistan for 13 million), while for some smaller countries the GPEI estimates lower numbers of children under 5 years old than the WPP (e.g., 2.2 million fewer for Thailand). Given uncertainty about the true population size and wastage [39] and to avoid underestimating vaccine needs compared to current practice, we apply a demographic uncertainty correction factor of 1.5 when estimating overall vaccine needs for SIAs, which implies effective wastage for all SIAs of w_sia_ = 1.2 × 1.5 = 1.8.

## Results

**Fig. 1:**
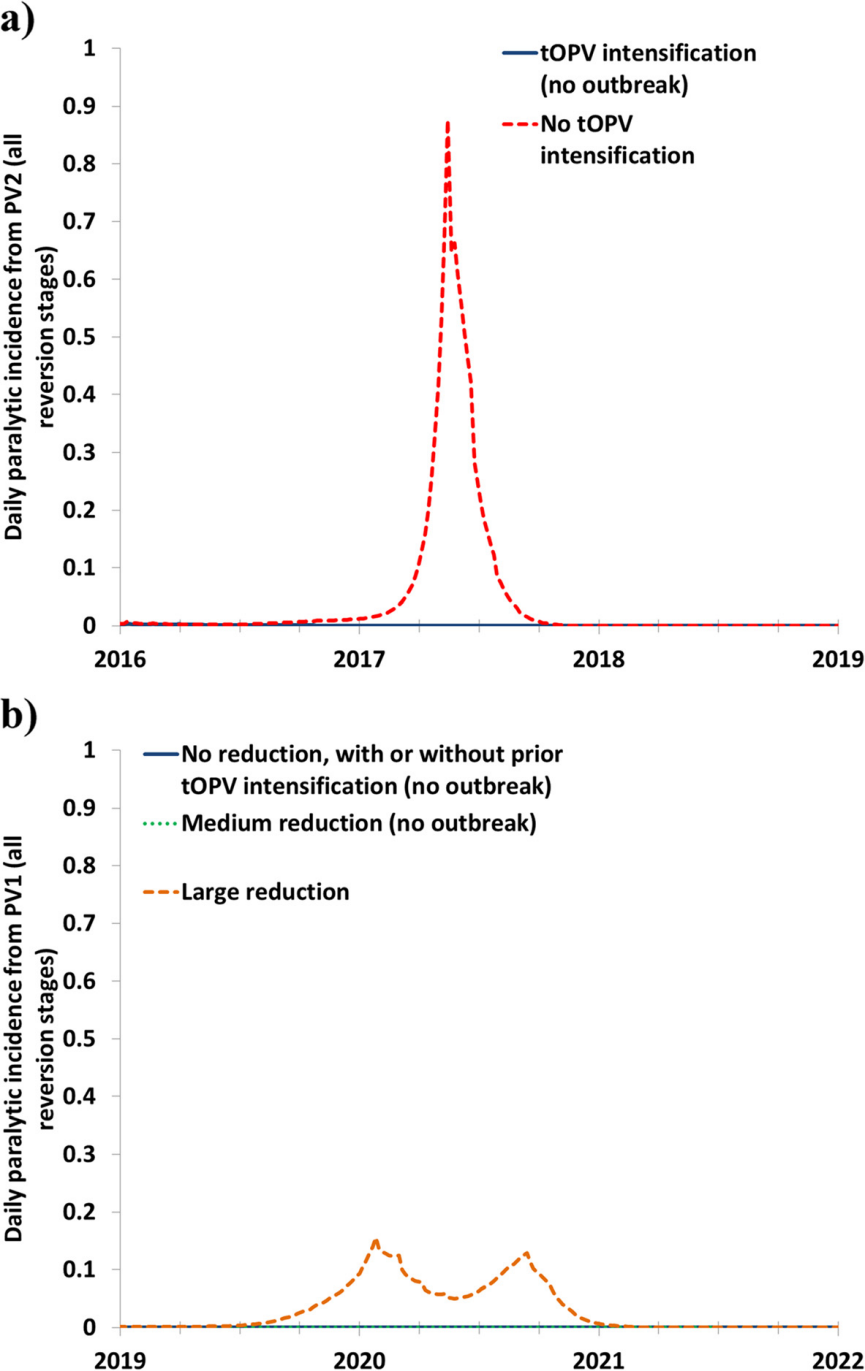
Impact of SIA intensity on cVDPV outbreaks after OPV2 cessation and OPV13 cessation showing the total paralytic incidence (i.e., including paralysis from OPV-related viruses in all reversion stages) in a block with a cVDPV outbreak in the event of insufficient homotypic OPV SIA. **a** Paralytic incidence due to serotype 2 polioviruses after OPV2 cessation in 2016, with or without tOPV intensification. **b** Paralytic incidence due to serotype 1 polioviruses after OPV13 cessation in 2019, for different scenarios of SIA frequency between January 1, 2017

Figure 1a shows the expected paralytic cases from serotype 2 polioviruses (PV2) for 2016-2019 with (solid blue curve) and without (red dashed curve) tOPV intensification prior to OPV2 cessation. The failure to intensify tOPV use prior to OPV2 cessation increases both the risk of missing the target date for OPV2 cessation, [30, 40] and the risk of cVDPV2 outbreaks after OPV2 cessation (Fig. 1a). Intensification of tOPV SIAs prior to OPV2 cessation prevents cVDPV2 outbreaks after OPV2 cessation such that the solid line becomes and remains 0 soon after OPV2 cessation. For the dashed curve without tOPV intensification, a cVDPV2 outbreak originates from a single subpopulation modeled as the under-vaccinated communities within one of the blocks representing the last reservoirs of WPV transmission (i.e., high R_0_, low RI coverage, poor SIA quality). Assumed aggressive response that involves block-wide SIAs [25] controls the outbreak in the subpopulation and prevents spread beyond it. The cVDPV2 outbreak in Fig. 1a results in over 50 expected paralytic cases and requires approximately 120 million filled mOPV2 doses from the outbreak response vaccine stockpile within the approximately 3.5-month duration of the outbreak response.

Figure 1b shows the expected paralytic cases from serotype 1 polioviruses (PV1) following OPV13 cessation for different options of SIA frequencies between January 1, 2017 and OPV13 cessation (Table 2). None of the options resulted in a serotype 3 cVDPV (cVDPV3) outbreak. The global model runs that assume no reduction in SIA frequency (solid blue curve) and medium reduction in SIA frequency (green dotted curve) prevent cVDPV1 outbreaks and thus become and remain 0 soon after OPV13 cessation. However, the global model run with a large reduction in SIA frequency (orange dashed curve) leads to paralytic cases associated with partially- and ultimately fully-reverted viruses derived from serotype 1-containing OPV vaccine that can continue to transmit in the context of insufficient population immunity at the time of OPV13 cessation. This occurs in the model in the same subpopulation that experienced a cVDPV2 outbreak with insufficient tOPV intensification. Figure 1b shows that the initial outbreak response (i.e., with 4 block-wide mOPV1 rounds) does not fully interrupt transmission. After some delay, a second peak in incidence occurs that triggers more outbreak response rounds (i.e., another 4 block-wide mOPV1 rounds) that stop the transmission. The cVDPV1 outbreak results in 5 expected paralytic cases and requires over 240 million filled mOPV1 doses from the outbreak response vaccine stockpile (i.e., 121 million doses during the first 4 rounds over a period of approximately 3.5 months and another 122 million doses beginning approximately 4 months later for the second 4 rounds, which also take approximately 3.5 months).

**Fig. 2:**
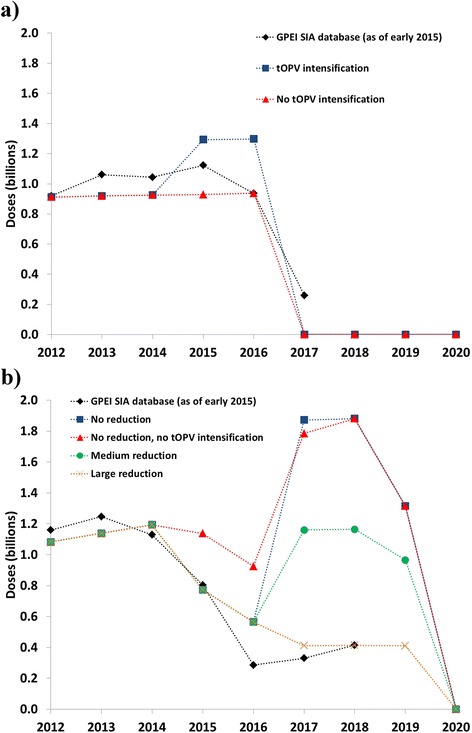
Estimated SIA vaccine needs from a global model [25] compared to recent GPEI plans. **a** tOPV needs. **b** bOPV needs

Figure 2 shows the estimated tOPV and bOPV SIA needs over time for the various scenarios, compared to the number of doses reported as required in the GPEI database. The GPEI SIA plan as of February 2015 recognizes some need to intensify tOPV use in SIAs before OPV2 cessation, as shown in Fig. 2a by the increase in expected tOPV needs in 2015 and the first quarter of 2016, before OPV2 cessation (Fig. 2a). However, intensification of tOPV use for SIAs in all modeled populations with low RI coverage (Table 1) requires a more pronounced increase in tOPV vaccine needs from approximately 930 million doses in 2014 to almost 1300 million doses in 2015 (i.e., a 40 % increase). Since the model takes advantage of using tOPV for SIAs in early 2016 before the OPV2 cessation date of April 1, 2016, we emphasize the importance of ensuring tOPV availability during this time.

Figure 2b shows the estimated bOPV needs. For 2012–2014, the model estimates similar bOPV needs for SIAs as the GPEI SIA database reports. For 2015, the bOPV needs remain similar between the current GPEI plans and the model estimates with tOPV intensification. However, without tOPV intensification, the global model uses more bOPV in 2015 than with tOPV intensification or with the current GPEI plans (Fig. 2b, consistent with the assumptions in Table 2), and less tOPV (Fig. 2a). The difference with or without tOPV intensification for 2017 reflects the aggressive mOPV2 use to respond to the cVDPV2 outbreak that occurs without tOPV intensification (Fig. 1a), which in the model overrides otherwise planned bOPV SIAs. From 2017 forward, the different bOPV use options result in substantial differences in bOPV needs. A large reduction in SIA frequency results in vaccine needs for 2017 and 2018 similar to the GPEI plans, but does not prevent cVDPV1 outbreaks (Fig. 1b). No reduction in SIA frequency results in an increase in bOPV needs because all SIAs that previously used tOPV switch to bOPV, but prevention of cVDPV1 and cVDPV3 outbreaks after OPV13 cessation does not require such high SIA intensity. A medium reduction avoids cVDPV1 outbreaks (Fig. 1b) and implies vaccine needs for 2017 and 2018 approximately halfway between no reduction in SIA frequency and the current GPEI plans.

Table 3 shows the model estimates of total expected tOPV and bOPV vaccine needs from RI and SIAs combined to manage cVDPV risks during the OPV cessation period. The global RI vaccine needs of approximately 620 million doses per year in the model significantly exceed the typical annual UNICEF projections for RI of approximately 235 million [41]. This occurs because UNICEF only procures a fraction of all OPV doses, with large self-producing countries (China, India), the Pan American Health Organization revolving fund, and vaccine demand from other countries accounting for the difference. Consequently, our modeling efforts suggest that the overall global tOPV needs amount to as much as 3.4 billion doses between January 1, 2015 and January 1, 2016. Based on the scenario of no reduction in SIA frequency after OPV2 cessation (not currently planned by the GPEI and not needed based on our results), bOPV needs remain very high up until OPV13 cessation, with a total of 8.0 billion doses between January 1, 2016 and April 1, 2019, and SIAs accounting for 71 % of the total. With a medium reduction in SIA frequency, the total bOPV needs during this time period decrease to 6.2 billion doses.

**Table 3:** Estimated total tOPV and bOPV vaccine needs based on the global model [25] with different assumptions about the type of vaccine used for SIAs and different SIA frequencies (not including vaccine needed for outbreak response activities) for options that prevent cVDPVs after OPV cessation^a^

Vaccine, time period	RI doses (billions)	SIAs doses (billions)	Total global needs (billions)
tOPV, January 1, 2015 –April 1, 2016	0.8	2.6	3.4
bOPV January 1, 2016 – April 1, 2019			
- No reduction in frequency	2.3	5.6	8.0
- Medium reduction in frequency	2.3	3.9	6.2

^a^The model assumes feasibility of OPV2 cessation in April 2016, and any delay in that date will add additional doses of tOPV

## Discussion

OPV cessation represents the only way to eliminate the risks associated with OPV use, [4, 42] but implementation comes with challenges and risks. Consistent with prior work, [13, 21, 40] this analysis suggests that high serotype-specific population immunity to transmission obtained through continued intense OPV use can prevent cVDPV outbreaks after OPV cessation of each serotype. The current GPEI plans provide a reassuring indication that tOPV use during SIAs will increase in 2015 and 2016 before the targeted OPV2 cessation date. However, our model suggests somewhat higher overall tOPV vaccine needs than currently planned to ensure high enough population immunity to transmission everywhere at the time of OPV2 cessation. Considering our results that suggest a cVDPV2 outbreak in only one modeled subpopulation without intensification, some may suggest the GPEI should target SIA intensification only in that subpopulation without tOPV intensification elsewhere. However, such a strategy appears highly imprudent in the context of uncertainty about true coverage, serotype-specific population immunity, OPV evolution, and extrapolation of the model to the real world [7, 25]. If cVDPV2 outbreaks occur, they would likely emerge in areas of historically poor immunization that would face challenges in achieving the aggressive outbreak response needed to contain the outbreak, which could jeopardize OPV2 cessation, in particular if other areas start with relatively lower population immunity to transmission due to insufficient intensification. Similarly, a strategy of minimal bOPV SIAs leading up to OPV13 cessation also leads to significant risk of undesirable cVDPV1 outbreaks after OPV13 cessation if any of the SIAs do not occur according to plan. Moreover, data on true RI and SIA coverage remain of poor quality in many settings, which means that minimal SIA schedules based on overestimates of true coverage would not provide high enough population immunity to transmission at OPV13 cessation to avoid cVDPVs. Due to longer reversion times and lower transmissibility of OPV1 and OPV3 viruses compared to OPV2, [7, 12] these viruses will die out sooner after OPV13 cessation than OPV2 viruses after OPV2 cessation, given the same level of population immunity to transmission. This suggests that after global WPV1 eradication and OPV2 cessation, the frequency of SIAs with bOPV could safely decrease to some extent. However, given the potential health consequences and stockpile vaccine needs in the event of cVDPV outbreaks of serotypes 1 or 3 after OPV13 cessation, more bOPV use than strictly needed before OPV13 cessation appears a necessary and prudent approach.

A challenge emerges in the context of perceived conflicting objectives of simultaneously ensuring WPV1 elimination and safe OPV2 cessation. Due to competition between the serotypes, tOPV leads to a lower individual first-dose take rate for serotype 1 than bOPV [12, 24, 33]. However, SIAs with tOPV do not decrease serotype 1 population immunity to transmission, and the difference between bOPV and tOPV SIAs remains very small, because with repeated tOPV doses reaching the majority of the population to induce serotype 2-immunity, subsequent doses become *de facto* bOPV doses with respect to serotype 1 and 3 take [26]. For example, a model exploring tOPV vs. bOPV SIA vaccine choices for northwest Nigeria found that using tOPV exclusively in all 11 SIAs between January 1, 2015 and OPV2 cessation would maintain high enough population immunity to prevent sustained WPV1 transmission and stop and prevent cVDPV2 transmission, while using bOPV for the rounds fails to stop cVDPV2 transmission [26]. Focusing on WPV1 alone to the detriment of serotype 2 population immunity (i.e., choosing bOPV over tOPV for SIAs) would effectively imply an acceptance of paralytic polio cases caused by cVDPV2 and a need to reconsider the current strategy for serotype-specific OPV cessation starting with OPV2 cessation [26]. Our results suggest the need for manufacturers to err on the side of producing more tOPV between now and the time of OPV2 cessation than they might expect based on current forecasts from the GPEI, although we recognize that they will need incentives to do so. Insufficient supplies of tOPV in the run-up to OPV2 cessation could create real threats to the OPV2 cessation timeline and cVDPV2 risk management efforts.

While the current GPEI plans appear to increase tOPV use before OPV2 cessation, they still create an expectation after OPV2 cessation and WPV eradication that the need for bOPV SIAs may drop significantly [2]. Our model suggests that such a decrease would lead to expected cVDPV1 outbreaks after OPV13 cessation. Thus, countries that use SIAs to maintain high population immunity to transmission and manufacturers that supply the vaccine for these SIAs should anticipate the need to continue to use bOPV for relatively frequent SIAs until OPV13 cessation.

While our model provides information that can support estimates of the global vaccine needs and considers the consequences of insufficient vaccine, we note several limitations. First, the model relies on a hypothetical characterization of the world that does not fully account for local heterogeneity (other than by age) within subpopulations of approximately 10 million people. In the context of forecasting vaccine needs, the GPEI plan focuses on a detailed characterization of this heterogeneity in coverage in some areas, which represents a complementary approach. Moreover, SIA plans may change based on the local epidemiological situation and global strategy and changes in RI. These factors complicate direct comparisons between the model results and GPEI plans, and explain some of the differences between modeled and actual historic vaccine needs during 2012-2015. The GPEI data further do not include all SIAs conducted outside of the scope of the GPEI (e.g., in China or Latin America), while our model includes all countries. However, exclusion of model blocks not included in the GPEI database only resulted in a moderate reduction of the global vaccine needs for SIAs (i.e., of approximately 10 %). Thus, the increase in tOPV needs associated with tOPV intensification would remain markedly steeper than that reflected in the GPEI estimates even if the GPEI estimates included those countries. Consistency between various data sources further limits the direct application of vaccine needs estimates from either source to accurately predict true demand. Furthermore, this analysis focuses on preventive SIAs prior to OPV cessation and does not comprehensively consider the needs for reactive outbreak response SIAs, which remains a priority for future research. The model also does not factor in the current move to use IPV co-administered with tOPV or bOPV in SIAs in some countries. While this strategy can overcome the issue of insufficient immunity provided by IPV in settings with low RI coverage and can take advantage of IPV’s ability to boost intestinal immunity of individuals with prior live poliovirus-induced immunity, it does not eliminate the issue of inherently limited protection from fecal-oral transmission provided by IPV-only. The effect of co-administering IPV with OPV in SIAs on transmission at the population level also remains unknown and requires further research, particularly in the context of the financial implications. Finally, all limitations and uncertainties from the global model carry through to this analysis [12, 25].

Despite its limitations, we hope that this analysis will stimulate further discussion about the immediate tOPV needs for successful OPV2 cessation, lead to recognition of the importance of sufficient OPV vaccine supplies during the polio endgame, and stress the importance of creating expectations and plans for bOPV use until OPV13 cessation. Our analysis also demonstrates a clear linkage and trade-off between using more OPV prior to OPV cessation and the amount of expected OPV needed in the stockpile for outbreak response. Insufficient use of OPV in preventive SIAs will lead to the need to shift more of the expected doses in the mOPV stockpiles available for outbreak response to filled doses instead of bulk, and could lead to greater ultimate OPV use if the outbreak response does not prevent the outbreak from spreading widely. Further research on outbreak response and vaccine needs for the associated mOPV stockpiles should consider these trade-offs.

## Conclusions

Managing the risks of cVDPVs in the polio endgame will require serotype-specific OPV SIAs in some areas prior to OPV cessation and lead to demands for additional doses of the vaccine in the short term that will affect managers and manufacturers.

## Abbreviations

bOPV: Bivalent OPV (serotypes 1 and 3)
cVDPV(1,2,3): Circulating vaccine-derived poliovirus (serotype 1, 2, or 3, respectively)
GPEI: Global Polio Eradication Initiative
IPV: Inactivated poliovirus vaccine
mOPV(1,2,3): Monovalent OPV (serotype 1, 2, or 3, respectively)
NID: National immunization day
OPV: Oral poliovirus vaccine
OPV(##) cessation: Globally-coordinated cessation of OPV containing the serotype(s) indicated by ##
oSIA: Outbreak response SIA
POL3: Coverage with 3 or more non-birth RI doses
pSIA: Planned, preventive SIA
PV(1,2,3): Poliovirus (of serotype 1, 2, or 3, respectively)
R_0_: Basic reproduction number
RI: Routine immunization
SIA: Supplemental immunization activity
SNID: Sub-national immunization day
tOPV: Trivalent OPV
VAPP: Vaccine-associated paralytic poliomyelitis
WPP: United Nations’ World Population Prospects
WPV(1,2,3): Wild poliovirus (serotype 1, 2, or 3, respectively)
